# Virtual reality stimulation for neuroprotection and neuroenhancement of vision in optic neuropathy patients: a prospective clinical trial

**DOI:** 10.1186/s12886-025-04576-w

**Published:** 2025-12-20

**Authors:** Katherine J. Healzer, Tasneem Z. Khatib, Gala Beykin, Zachary Wennberg-Smith, Mariana Nuñez, Andrew D. Huberman, Jeffrey L. Goldberg

**Affiliations:** 1 Department of Ophthalmology, Spencer Center for Vision Research, Byers Eye Institute, Stanford University School of Medicine, 2452 Watson Court, Palo Alto, CA 94303 USA; 2 Department of Clinical Neurosciences, University of Cambridge, Cambridge, UK; 3 Department of Ophthalmology, Cambridge University Hospitals, Cambridge, UK; 4 Department of Neurobiology, Stanford University School of Medicine, Stanford, CA USA

**Keywords:** Glaucoma, Visual stimulation, Visual protection, Visual enhancement

## Abstract

**Background:**

This study aimed to assess the safety and efficacy of virtual reality (VR) visual stimulation on neuroprotection and neuroenhancement in optic neuropathy patients.

**Methods:**

Open-label, prospective, phase I clinical trial, enrolling 21 participants with glaucoma, plus 1 patient with ischemic optic neuropathy after hydrocephalus. Participants received commercially available VR headsets (HTC Vive or Oculus Rift) for home use loaded with a study-specific visual stimulus program. The VR visual stimulation protocol consisted of up to 1-hour daily sessions, 5 days per week, for 12-week cycles repeatable for up to 24 months. Safety was assessed through adverse event monitoring, and compliance was assessed through self-report. Testing of function (visual acuity and visual field) and structure (optical coherence tomography (OCT) for retinal nerve fiber layer (RNFL) and ganglion cell complex (GCC) thickness) were measured at baseline and follow-up.

**Results:**

VR-based visual stimulation was safe and well tolerated across the 22-patient cohort. Mean baseline intraocular pressure (IOP) was 12.06 ± 0.76 mmHg and there was no significant change in IOP at follow-up. 8 of 22 patients were sufficiently compliant with use to analyze for efficacy measures. Analysis of Humphrey visual field (HVF) mean deviation (MD) of individual patients revealed that two eyes of two patients showed improvement and seven eyes of five patients showed worsening during the study duration. One patient using Goldmann visual fields (GVF) demonstrated improvement in sensitivity in both eyes. There were no statistically significant changes in visual acuity, HVF MD, GCC thickness, or RNFL thickness across the full patient population. Two illustrative cases were selected to demonstrate the potential therapeutic effects of VR visual stimulation.

**Conclusion:**

These findings demonstrate that VR-based visual stimulation is safe, well-tolerated, and may provide functional benefits in selected patients. Additional studies are warranted to further investigate this therapeutic approach.

**Trial registration:**

ClinicalTrials.gov, TRN: NCT07071129, Registration date: 9 July 2025, retrospectively registered.

**Supplementary Information:**

The online version contains supplementary material available at 10.1186/s12886-025-04576-w.

## Background

Glaucoma is the leading cause of irreversible blindness in the world [1]. It represents a neurodegenerative disease characterized by the gradual demise and ultimate death of retinal ganglion cells (RGCs) located in the inner retina [2]. Current approaches to treat glaucoma rely on reducing intraocular pressure (IOP) through surgical and medical interventions to slow progression of the disease [3]. However, many patients still experience progression despite successfully decreasing their IOP [4–7]. Furthermore, there are no approved treatments available that target the RGCs and optic nerve directly for neuroprotection or neuroenhancement [3], making this a major goal in glaucoma research.

Potential treatments that directly target RGC survival (i.e. neuroprotection) and function (i.e. neuroenhancement) in glaucoma have gained considerable attention. Recent advances in glaucomatous neurodegeneration have identified several potential therapeutic targets [3, 8], including providing neurotrophic factors or increasing physiological levels of electrical activity [9–12]. Neurotrophic factors including ciliary neurotrophic factor (CNTF) delivered by encapsulated cell therapy in an intravitreal implant [13, 14] and nerve growth factor (NGF) delivered as an eye drop have been tested in patients with glaucoma and other optic neuropathies with early signals of neuroenhancement measured by structure and function [11, 12, 15–18]. Electrical activity in RGCs can promote neurotrophic factor responsiveness and stimulate other pathways that promote neuroprotection or regeneration [19, 20]. In human trials, increasing electrical activity through direct electrical stimulation has shown statistically significant positive effects on visual field testing in patients with glaucoma [21–23].

Another way to stimulate activity in RGCs may be through visual stimulation, which has emerged as a promising non-invasive intervention based on compelling preclinical and clinical evidence. In pre-clinical experiments, high-contrast visual stimulation has been shown to promote axon regeneration in RGCs following optic nerve injury in rodent models, albeit with an even greater effect in combination with stimulating a growth-promoting signaling pathway by gene therapy [24]. The neuroprotective effects were mediated by increased RGC spiking activity driven by visual stimulation, suggesting that controlled visual input could serve as a powerful therapeutic modality.

In clinical settings, early evidence also points to therapeutic potential of visual stimulation, with one small clinical study demonstrating a statistically significant improvement in visual defects among glaucoma patients who utilized virtual reality (VR)-based visual stimulation [25]. VR technology offers unique advantages for delivering controlled visual stimulation protocols. VR systems provide immersive, standardized visual environments that can be precisely controlled and customized for individual patients [26]. The portability of modern VR headsets enables home-based therapy, thereby improving patient accessibility to modalities that might include diagnosis, monitoring, and/or treatment by reducing the burden of frequent clinic visits. Additionally, VR platforms can deliver complex visual stimuli designed to maximize RGC activation while monitoring usage patterns and treatment adherence.

Building on this foundation of preclinical and early clinical evidence, we hypothesized that structured VR visual stimulation could provide neuroprotective or neuroenhancing effects in patients with glaucoma or other optic neuropathies. Here, we evaluated the efficacy of a single visual stimulus delivered by VR on retinal structure and visual function in a prospective, open-label trial that included patient eyes with CNTF-secreting implants.

## Methods

### Study design

This was a prospective, open-label, single-arm clinical trial conducted at the Byers Eye Institute, Stanford University. The study was approved by the Stanford Institutional Review Board and registered at ClinicalTrials.gov (NCT07071129). All participants provided written informed consent prior to enrollment.

Eligible participants were aged 12 years or older with glaucoma, other retinal diseases, or disorders of the visual system. Key inclusion criteria included: (1) best-corrected visual acuity of 20/200 or better in at least one eye or ability to see visual stimuli in at least one eye; (2) sufficient fixation ability; (3) ability to comply with study procedures and visits; and (4) ability to provide informed consent. Exclusion criteria included: (1) electric or electronic implants (such as cardiac pacemakers); (2) metal artifacts in the head or trunk area (except dental implants); (3) epilepsy or photosensitivity; (4) autoimmune diseases; (5) acute conjunctivitis; or (6) nystagmus. Patients who were in prior clinical trials were allowed to be enrolled.

#### Visual stimulation

Participants received commercially available VR headsets (HTC Vive or Oculus Rift) for home use. The VR visual stimulation protocol consisted of up to 1-hour daily sessions, 5 days per week, for 12-week cycles.

**Fig. 1:**
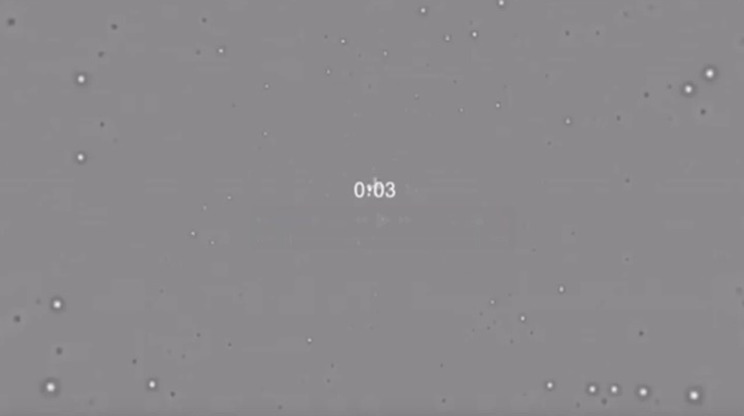
Visual stimulation program

The VR visual stimulation was designed as a computational model developed to maximize RGC activity with high-contrast visual patterns and moving stimuli, while minimizing motion sickness and discomfort [Figure 1 and Supplemental Movie 1]. Participants performed VR sessions while seated under supervision during initial training in the clinic and continued therapy at home.

Primary outcome measures included: (1) Humphrey visual field (HVF) mean deviation (MD) and visual field index (VFI) testing using the 24 − 2 SITA Standard Swedish Interactive Threshold Algorithm every 12 weeks; (2) best-corrected visual acuity by ETDRS charts at each visit; and (3) optical coherence tomography (OCT) for retinal nerve fiber layer (RNFL) and ganglion cell complex (GCC) thickness. Secondary measures included IOP measurements and safety assessments including adverse event monitoring.

### Data collection and analysis

Data quality underwent rigorous evaluation, including the exclusion of HVF data with fixation losses, false negatives or false positives > 20%. OCT images extracted by certified technicians underwent quality review, and scans were excluded for a signal strength of 4 or less, segmentation failure of the algorithms, overall poor scan quality, or an incomplete scan. Descriptive statistics were used as noted through the results section.

## Results

**Fig. 2:**
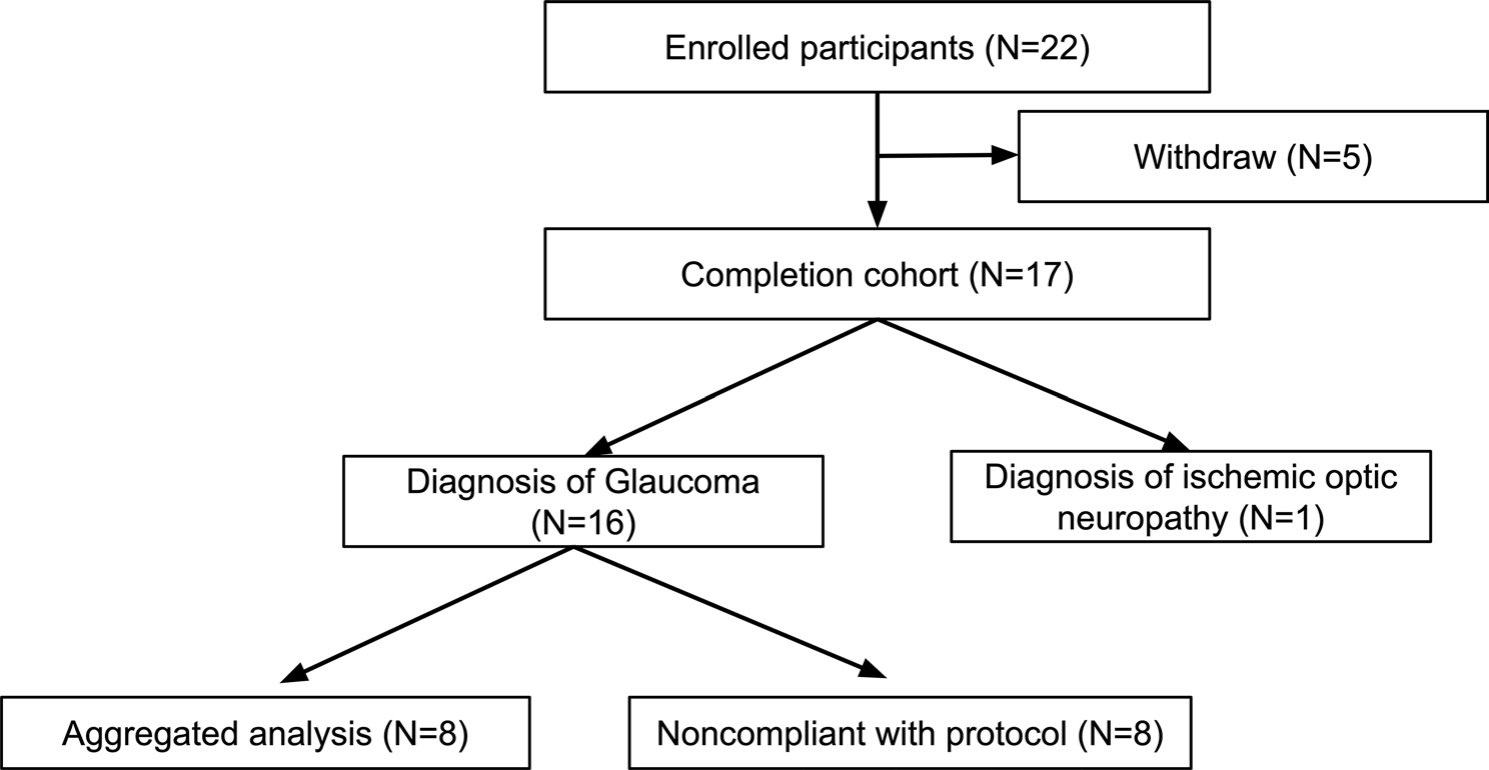
Participant flow chart

A total of 22 patients were enrolled in the study from April 2018 to February 2020. Comprehensive participant and eye baseline characteristics were tabulated [Table 1]. Two patients withdrew voluntarily, one due to time constraints, and one due to not being able to operate the software in the headset. Three patients terminated use due to an adverse event, one due to increased temporomandibular joint pain (TMJ) after use, one due to described overstimulation upon device use, and one due to anxiety upon increasing awareness of their scotoma. Of the remaining 16 glaucoma patients and 1 ischemic optic neuropathy patient, 8 glaucoma patients were excluded from aggregate analysis after reporting low or stopping usage of the VR visual stimulation [Figure 2]. Six eyes of six patients had received an intraocular high-dose CNTF-secreting implant (NT-501, now FDA-approved as Revakinagene Taroretcel-lwey; Neurotech, Cumberland, RI, USA), prior to initiating VR stimulation.

**Table 1:** Baseline patient and eye characteristics

Characteristics		Patients
Sex		
	Male	11 (50%)
	Female	11 (50%)
Race		
	White	16 (73%)
	African American	1 (5%)
	Asian	5 (23%)
Age		
	Mean	63
	Median	66.5
	Range	16-82
Diagnosis		
	Primary Open Angle Glaucoma	18
	Pseudoexfoliation Glaucoma	1
	Pigmentary Glaucoma	1
	Glaucoma Suspect	1
	Other	1
Disease Severity		
	Mild (HVF MD > -6 dB)	2
	Moderate (-6 dB ≤ HVF MD ≥ -12 dB)	3
	Severe (HVF MD < -12 dB)	16
Glaucoma Medication		
Mean Drops per Patient		2.38
Median Drops per Patient		2
Medication Class		
	Prostaglandin Analog	18
	Beta-blocker	8
	α-2 Adrenergic agonist	6
	Carbonic anhydrase inhibitor	19
	Rho kinase inhibitor	4
Previous CNTF-Secreting Implant		6 eyes

### Intraocular pressure

**Fig. 3:**
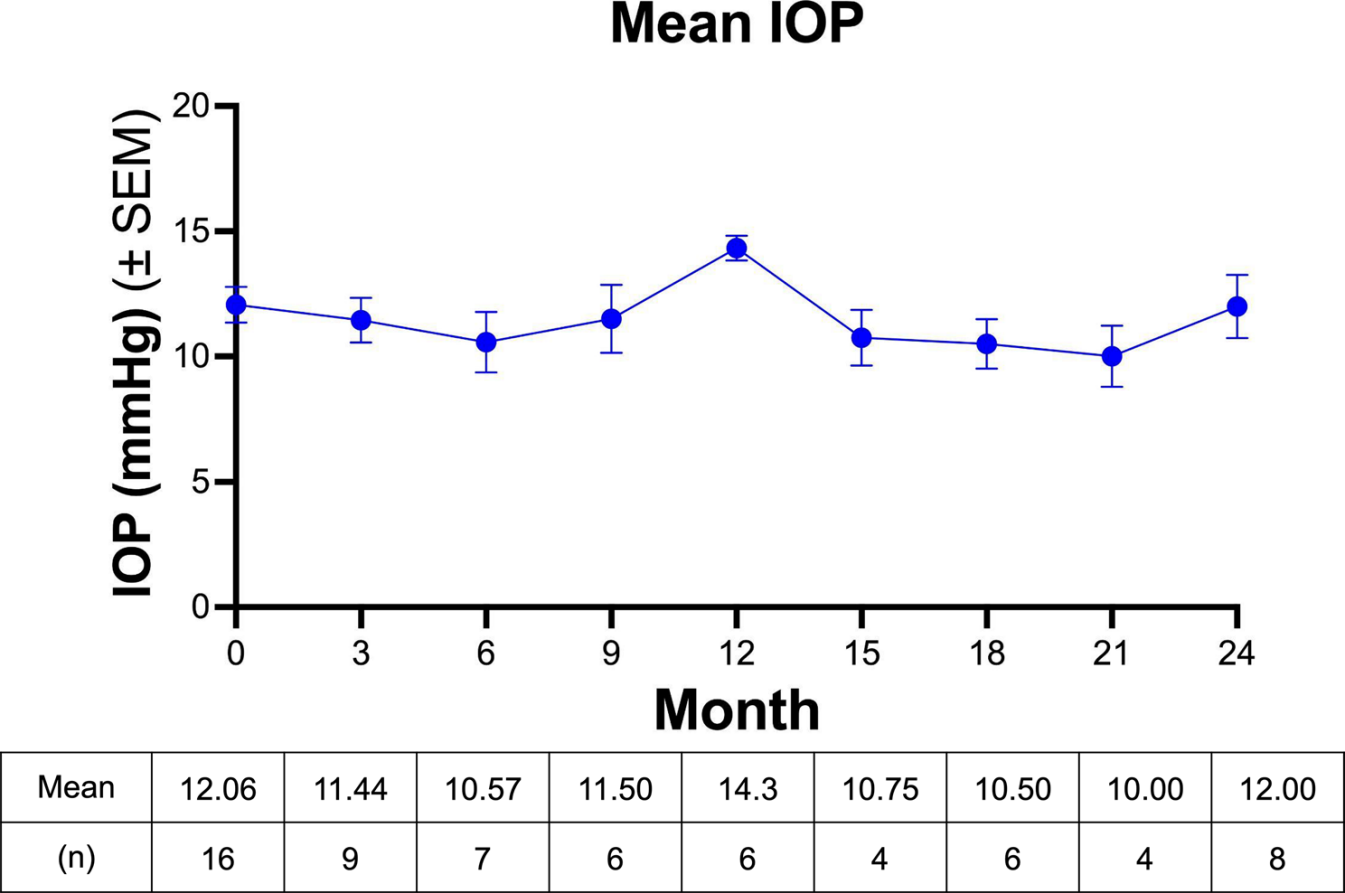
No change in intraocular pressure after VR Stimulation. Mean IOP at baseline to month 24. N represents the number of eyes analyzed at each time point

IOP measurements remained stable throughout the study period. No significant changes in IOP were observed during VR therapy, and no participants required modification of their glaucoma medications during the study period [Figure 3].

### Visual acuity

**Fig. 4:**
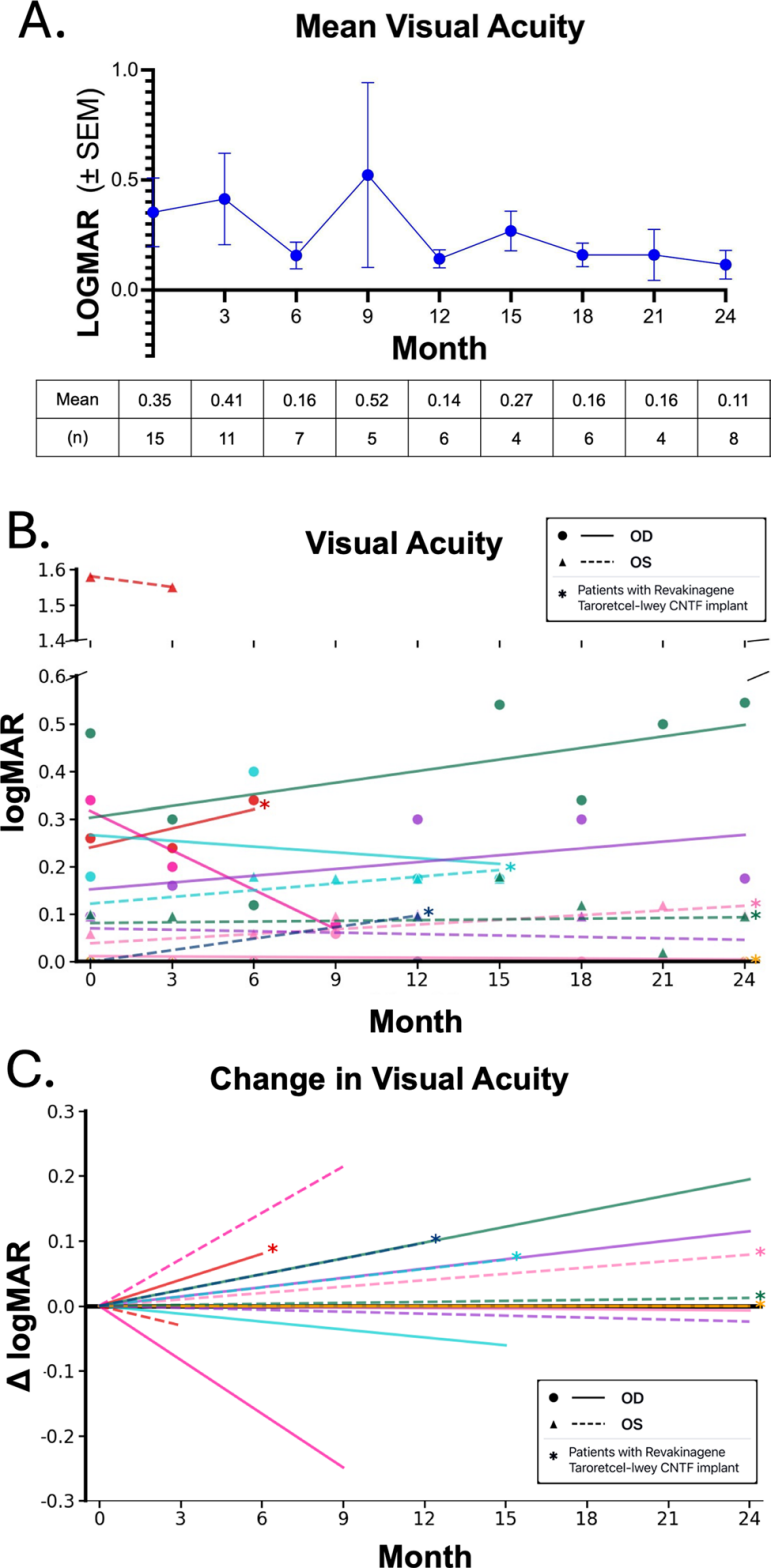
No changes in best corrected visual acuity (LOGMAR scores) throughout the treatment course. **A**. Mean Best Corrected Visual Acuity (BCVA) and number (n) at each time point. **B**. Change in BCVA per patient; not graphed is one patient who had count fingers vision at 0 and 9 months. Asterisks denote patients who had received a Revakinagene Taroretcel-lwey CNTF implant prior to initiating VR stimulation. **C**. Change in BCVA against baseline vision

Central visual acuity remained stable with no significant net change [Figure 4A]. One patient’s visual acuity improved by 13 letters, and two patients’ visual acuity declined, one from count fingers at 5” to hand motion, and the other improved by 16 letters in 6 months and declined by 21 letters to the end of the study period [Figure 4B, C].

### Visual field outcomes

**Fig. 5:**
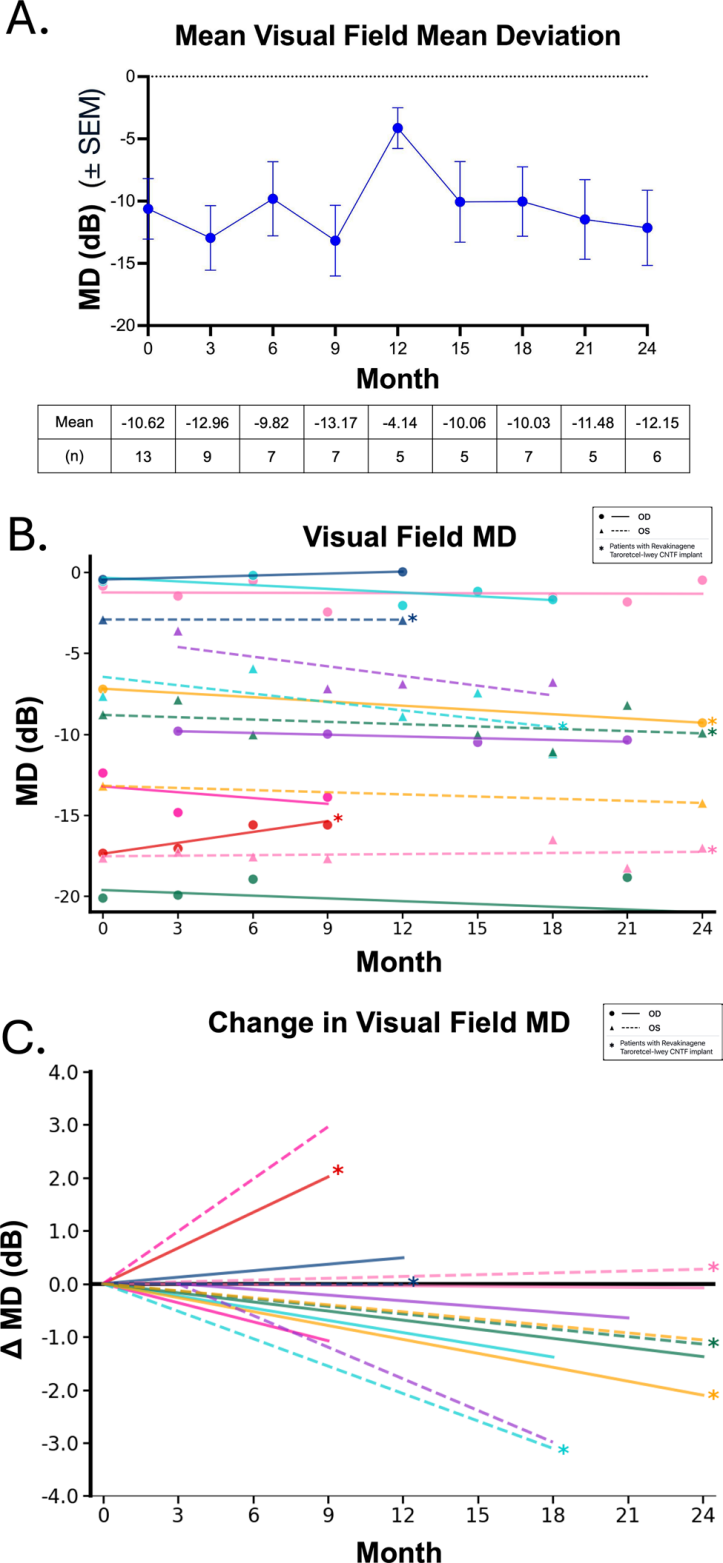
Visual Field Outcomes during VR Stimulation. **A**. HVF mean deviation (MD ± SEM) and number (n) at each time point during VR stimulation. **B**. Visual field trends from baseline per patient. Each line represents the trendline of HVF MD per eye per patient. Asterisks denote eyes who had received a Revakinagene Taroretcel-lwey CNTF implant prior to initiating VR stimulation. **C**. Visual field change from baseline per patient

Aggregating the HVF measurements for the 8 participants with glaucoma who used the VR device regularly, there was no net change in MD between baseline and month 24 [Figure 5A]. Upon evaluating individual patients and using at least 1 dB as a threshold for change, two eyes of two patients showed improvement and seven eyes of five patients showed worsening during the study duration [Figure 5B, C]. One patient using Goldmann visual fields (GVF) demonstrated improvement in sensitivity in both eyes. One patient who demonstrated HVF improvement had received a Revakinagene Taroretcel-lwey CNTF implant two years prior to initiating VR stimulation.

### Structural outcomes

**Fig. 6:**
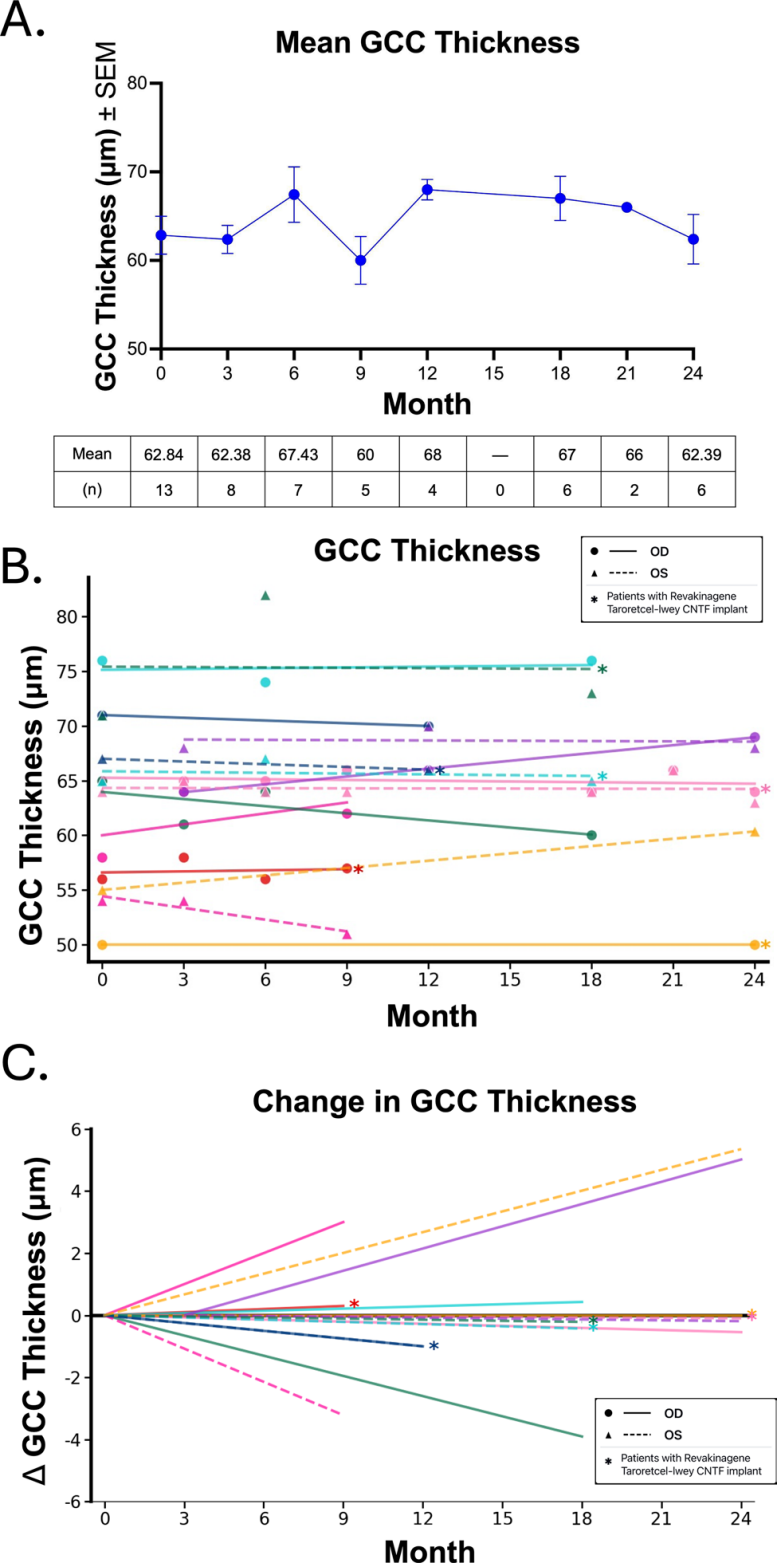
GCC Thickness during VR Stimulation. **A**. Mean GCC thickness ± SEM and number (n) at each time point during VR stimulation. **B**. GCC trends from baseline per patient. Asterisks denote eyes who had received a Revakinagene Taroretcel-lwey CNTF implant prior to initiating VR stimulation. **C**. GCC change from baseline per patient

**Fig. 7:**
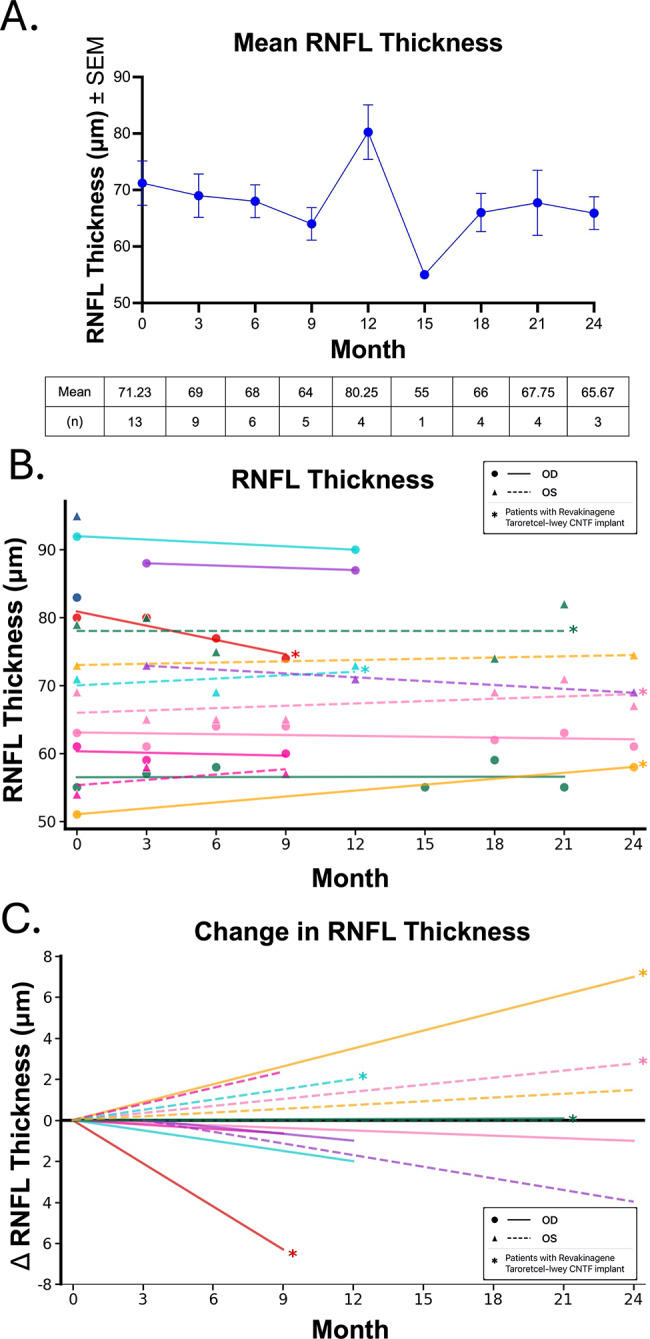
RNFL Thickness during VR Stimulation. **A**. Mean RNFL thickness ± SEM and number (n) at each time point during VR stimulation. **B**. RNFL trends from baseline per patient. Asterisks denote eyes who had received a Revakinagene Taroretcel-lwey CNTF implant prior to initiating VR stimulation. **C**. RNFL change from baseline per patient

OCT measurements of RNFL and GCC thickness were obtained at baseline and follow-up visits. No significant changes in average retinal structure were detected in the overall cohort in either GCC [Figure 6A] or RNFL [Figure 7A]. For individual patients, the GCC and RNFL trend revealed individual variations. No patients experienced a change in GCC thickness more than 5 μm [Figure 6B, C] during the study duration. One patient with a Revakinagene Taroretcel-lwey CNTF implant demonstrated an increase in RNFL thickness more than 5 μm and one patient with a Revakinagene Taroretcel-lwey CNTF implant demonstrated a decrease in RNFL thickness more than 5 μm [Figure 7B, C]. Because there was no significant change in the individual measures, we did not analyze for any correlation between changes in HVF or RNFL or GCC.

### Case studies of responsive patients

To illustrate the potential therapeutic effects of VR visual stimulation, we present detailed case studies of two patients who used the device consistently and demonstrated visual field improvement.

#### Patient A

**Fig. 8:**
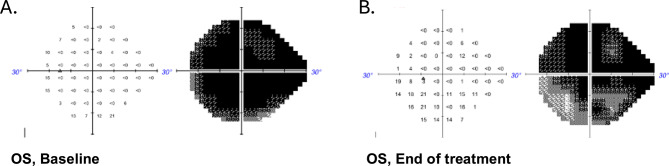
Patient A Humphrey Visual Field OS at Baseline and at End of Treatment Period. **A**. Baseline visual field demonstrated severe defects. **B**. Visual field demonstrated functional improvement over the treatment period

Patient A is a 75-year-old man diagnosed with severe-stage pseudoexfoliation glaucoma. HVF of the right eye was excluded due to high false positives (> 20%). Visual acuity was CF at 5’’ in the left eye. At baseline, the patient was using two topical glaucoma medications including a beta-blocker and prostaglandin analogue on top of a previous trabeculectomy. IOP measured 16 mmHg. OCT revealed retinal nerve fiber layer thickness of 54 μm. The baseline visual field demonstrated severe defects with a HVF MD of -29.11 dB and visual field index (VFI) of 5%. This patient demonstrated high compliance with the VR visual stimulation protocol, and no adverse events related to VR use were documented. The left eye showed improvement over the treatment period, with HVF MD of -25.50 dB, VFI of 13% at 9 months [Figure 8]. He reported noticeable improvement in his vision in his left eye.

#### Patient B

**Fig. 9:**
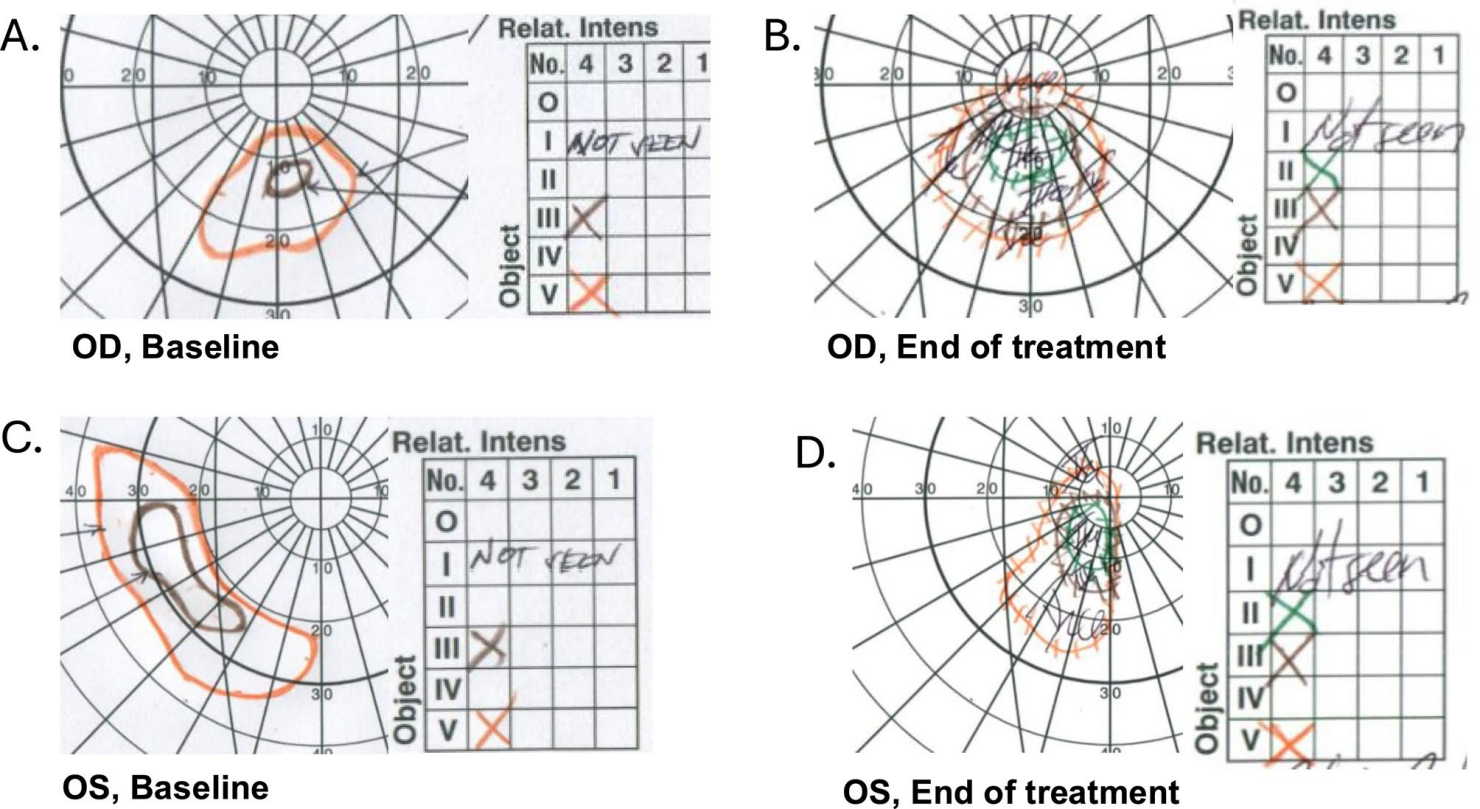
Patient B Humphrey Visual Field OU at Baseline and at End of Treatment Period. **A**, **C**. Baseline GVF demonstrated severe defects. **B**, **D**. Both eyes demonstrated functional improvement over the treatment period with higher sensitivity in GVF

Patient B is a 16-year-old woman previously diagnosed with hypertensive hydrocephalus at age 13. This patient had lost significant vision with severe optic nerve head edema and subsequent atrophy bilaterally and had no cognitive or mental health deficits by report or by clinical observation over the length of the study. At baseline, the patient was using no ocular medication and had no history of ocular surgery. Her BCVA was 20/400 OD and 20/600 OS. IOP measured 13 mmHg OD and 14 mmHg OS. OCT was not interpretable due to poor fixation. This patient demonstrated high compliance with the VR visual stimulation protocol, and no adverse events related to VR use were documented. Both eyes demonstrated functional improvement over the treatment period; the left eye improved three letters on ETDRS. The left and right eyes had increased sensitivity on GVF. The patient experienced a subjective reduction in central visual haze affecting both eyes and noticed more peripheral vision in the right eye [Figure 9].

## Discussion

This study presents an open-label, prospective pilot trial evaluating VR visual stimulation as a potential neuroprotective or neuroenhancing therapy, enrolling primarily glaucoma patients. Our findings demonstrate that VR-based visual stimulation is safe, well-tolerated, and may provide functional benefits in selected patients.

The well-tolerated safety profile observed in this study is consistent with the widespread use of commercial VR systems and supports the feasibility of VR therapy in ophthalmic applications and in clinical practice. However, three patients discontinued use due to adverse events: (1) TMJ pain after device use, (2) subjective sensory overstimulation upon device use, and (3) anxiety upon increasing awareness of their scotoma. Although there is sparse literature examining the link between VR headsets and temporomandibular disorders, there are measurable changes in muscle pain and discomfort with VR headset use. Specifically, there is direct cervical spine loading from headset weight, and some users may experience strained neck postures and elevated muscle activity [27]. In addition, cybersickness is a known side effect of VR, and can limit use by affected patients [28, 29]. There is also documentation of unfamiliar or ambiguous patterns of sensory stimulation causing adverse effects impacting the vestibular system, potentially contributing to dizziness or vertigo [30]. Finally, the psychological burden of glaucoma is well established [31], and glaucoma patients are about 6 times more likely to experience depression and 3 times more likely to experience anxiety [32]. Increased anxiety and depression may contribute to disease progression, as RNFL thinning rate and disc hemorrhage are associated with anxiety, and HVF MD is associated with depression [33]. Although not included in this trial, baseline psychological assessments might reasonably be included in future work.

Furthermore, 8 other patients who enrolled in the study subsequently reported not having time or motivation to keep up with using the VR headset during the trial, limiting the analyzable trial data set to 8 patients with glaucoma and 1 with ischemic optic neuropathy. This lack of adherence is consistent with other measures of adherence in glaucoma patients, e.g. with ocular medication, as up to 50% of patients do not receive the intended benefits of medication due to noncompliance [34]. VR has further challenges, as it requires a level of technical proficiency [35]. Although current models are more user-friendly, the use of a head-mounted display can be problematic. Furthermore, some head-mounted displays can have a cable tether, which can create a risk of tripping for some patients. The game model and 3D-rendered world of many VR therapies can be challenging for elderly patients, especially those who did not grow up with computer games. The patients who have limited video game literacy may find elements of VR frustrating. On the other hand, the home-based nature of VR therapy offers significant advantages in terms of patient convenience and healthcare resource utilization. Patients can perform therapy sessions at their convenience while maintaining regular monitoring through telemedicine and periodic clinic visits. More research is needed to understand how to make VR more user-friendly for elderly patients and those with visual impairment, as the market is dominated by younger consumers, and computer-based over-the-internet solutions not relying on VR headset purchase and maintenance are also providing opportunities to reach patients at home [36].

The visual field trend analysis across this cohort revealed patterns that suggest potential therapeutic effects of VR stimulation for some patients. As this was not a randomized control trial, this remains speculative and cannot be disentangled from the natural disease course, or placebo (change in symptoms) or Hawthorne (change in behavior) effects. Although these changes were not statistically significant across all treated patients during the duration of the study, four eyes of three patients demonstrated improvement detectable on visual field testing and in some cases by the patients themselves. Due to the case report level of these examples, we would not conclude that there is a reliable efficacy signal here, but these findings do align with preclinical studies demonstrating activity-dependent neuroprotection and enhancement in RGCs [24]. The mechanism likely involves visual stimulation-induced increases in RGC activity, which can promote cell survival, enhance synaptic function, and potentially facilitate axon regeneration [37]. The potential for VR visual stimulation to slow or reverse glaucomatous progression represents a paradigm shift in adjuvant glaucoma management. Unlike current therapies that focus solely on IOP reduction, visual stimulation directly targets the neural components of the disease. This approach could be particularly valuable for patients with normal-tension glaucoma or those who continue to progress despite adequate IOP control. Alternatively, direct electrical stimulation could be substituted for visual stimulation. Clinical trials in glaucoma patients have shown significant improvement of a visual field deficit in addition to significantly improved temporal processing of visual stimuli, detection performance in static perimetry, and visual acuity after alternating current electrical stimulation for 40 min for 10 days [21]. These visual changes were stable for at least 2 months. Thus, visual or electrical stimulation remain strong candidate therapies for glaucoma and other optic neuropathies.

Pre-clinical data also suggests that visual or electrical stimulation together with growth factor signaling together have synergistic effects [9, 24]. This small sample size of patients with a Revakinagene Taroretcel-lwey CNTF implant and completing the VR visual stimulation protocol also did not show significant improvement. Combination approaches incorporating visual or electrical stimulation with other neuroprotective strategies may merit further exploration.

Several limitations should be acknowledged. Most importantly, the single-arm design without a randomization or control group limits our ability to definitively attribute observed changes to VR therapy rather than natural disease variation or regression to the mean. The relatively small sample size, variable compliance patterns, and self-report monitoring of device use and adverse events limit the statistical power for detecting treatment effects. Furthermore, while safety and tolerability findings reflect data from all 22 enrolled participants, efficacy outcomes were limited to the 8 patients who reported higher compliance with the protocol. This introduces potential compliance bias, which may limit the generalizability of these findings. In a future randomized control trial, such compliance bias would be expected to distribute more evenly between treatment and control groups. Thus, these data should be treated as a case series to not overemphasize the observed effects.

Equally limiting to conclusions is that the visual stimulus used in this study may not be optimal for stimulating RGCs or RGC function, and without confirmatory testing of the stimulus, e.g. with visually evoked potentials (VEP or pattern electroretinogram (PERG)) testing, we cannot be certain how much visual stimulation was provided. The mammalian retina contains many distinct RGC subtypes, each with specialized properties and stimulus preferences [38–40]. This study builds on previous research and used a unique visual stimulus with high-contrast black and white patterns with motion components which may preferentially activate direction-selective ganglion cells that respond strongly to movement in a preferred direction [41]. The functional assay of static perimetry did not then test this RGC subset as an outcome measure. Additionally, computational studies have demonstrated that RGC subtypes exhibit frequency-dependent responsiveness, with small bistratified cells responding preferentially to high-frequency stimulation and potentially contributing to blue-yellow color processing [42]. Frequency-specific visual stimulation paradigms may similarly target distinct RGC populations based on their electrophysiological properties. Stimulating and then testing different RGC subtypes that respond uniquely to visual features such as direction, motion, contrast, and color may provide insights into which RGCs are damaged and are most responsive to visual stimulation to provide neuroprotection or neuroenhancement [42]. Furthermore, recent research demonstrates that different RGC subtypes exhibit varying degrees of vulnerability to glaucomatous damage [43], and future visual stimulation studies may allow for personalized stimulations to create targeted therapy and optimal stimuli for subgroups of RGCs—again, perhaps using VEP or PERG as a biomarker for stimulus delivery and effect size. The development of more sophisticated VR platforms with integrated monitoring capabilities could enhance both treatment delivery and compliance assessment. Artificial intelligence algorithms could potentially personalize stimulation protocols based on individual patient responses and disease characteristics. Investigation of optimal stimulation parameters, including session duration, frequency, and visual pattern characteristics, will be important for maximizing therapeutic effects.

## Conclusion

This pilot study demonstrates that VR visual stimulation is a safe and potentially effective therapy for patients with glaucoma. Although there was no detectable effect measured across the population, including in the subset of eyes with a CNTF-secreting implant, the favorable safety profile, combined with evidence of functional improvement in visual field parameters in eyes of some compliant users, supports further investigation and optimization of this novel therapeutic approach. These preliminary results support the development of visual stimuli with definitive stimulatory effects, and then larger, longer, randomized controlled trials to definitively establish the efficacy of VR visual stimulation in glaucoma.

## Supplementary Information

Below is the link to the electronic supplementary material.

Supplementary Material 1

## Data Availability

Data availability is restricted in this study as participant consent and IRB approval did not authorize public data sharing, but limited deidentified data may be available upon contacting the corresponding author.

## Abbreviations

VR: Virtual reality
OCT: Optical coherence tomography
RNFL: Retinal nerve fiber layer
GCC: Ganglion cell complex
IOP: Intraocular pressure
HVF: Humphrey visual field
MD: Mean deviation
GVF: Goldmann visual field
RGC: Retinal ganglion cell
CNTF: Ciliary neurotrophic factor
NGF: Nerve growth factor
TMJ: Temporomandibular joint
VFI: Visual field index
VEP: Visually evoked potentials
PERG: Pattern electroretinogram
